# Culture in Glucose-Depleted Medium Supplemented with Fatty Acid and 3,3′,5-Triiodo-l-Thyronine Facilitates Purification and Maturation of Human Pluripotent Stem Cell-Derived Cardiomyocytes

**DOI:** 10.3389/fendo.2017.00253

**Published:** 2017-10-09

**Authors:** Bin Lin, Xianming Lin, Maxine Stachel, Elisha Wang, Yumei Luo, Joshua Lader, Xiaofang Sun, Mario Delmar, Lei Bu

**Affiliations:** ^1^Leon H. Charney Division of Cardiology, New York University School of Medicine, New York, NY, United States; ^2^Key Laboratory for Major Obstetric Diseases of Guangdong Province, Key Laboratory of Reproduction and Genetics of Guangdong Higher Education Institutes, The Third Affiliated Hospital of Guangzhou Medical University, Guangzhou, China; ^3^Department of Cell Biology, The Helen L. and Martin S. Kimmel Center for Stem Cell Biology, New York University School of Medicine, New York, NY, United States

**Keywords:** human pluripotent stem cell-derived cardiomyocytes, purification, maturation, fatty acid, 3,3′,5-triiodo-l-thyronine, electrophysiological characteristics

## Abstract

With recent advances in stem cell technology, it is becoming efficient to differentiate human pluripotent stem cells (hPSCs) into cardiomyocytes, which can subsequently be used for myriad purposes, ranging from interrogating mechanisms of cardiovascular disease, developing novel cellular therapeutic approaches, as well as assessing the cardiac safety profile of compounds. However, the relative inability to acquire abundant pure and mature cardiomyocytes still hinders these applications. Recently, it was reported that glucose-depleted culture medium supplemented with lactate can facilitate purification of hPSC-derived cardiomyocytes. Here, we report that fatty acid as a lactate replacement has not only a similar purification effect but also improves the electrophysiological characteristics of hPSC-derived cardiomyocytes. Glucose-depleted culture medium supplemented with fatty acid and 3,3′,5-Triiodo-l-thyronine (T_3_) was used during enrichment of hPSC-derived cardiomyocytes. Compared to untreated control cells, the treated cardiomyocytes exhibited enhanced action potential (AP) maximum upstroke velocity (as shown by a significant increase in dV/dt_max_), action potential amplitude, as well as AP duration at 50% (APD_50_) and 90% (APD_90_) of repolarization. The treated cardiomyocytes displayed higher sensitivity to isoproterenol, more organized sarcomeric structures, and lower proliferative activity. Expression profiling showed that various ion channel and cardiac-specific genes were elevated as well. Our results suggest that the use of fatty acid and T_3_ can facilitate purification and maturation of hPSC-derived cardiomyocytes.

## Introduction

Recent developments in stem cell technology hold promise for use in clinical applications. In the cardiovascular field, new differentiation methods now enable rapid and efficient generation of human pluripotent stem cell (hPSC)-derived cardiomyocytes (1, 2), which can subsequently be utilized as a powerful platform for cardiovascular disease modeling and moderate- to high-throughput evaluation of drug safety profiles (3). One caveat to the accuracy of such testing is the interference from other cells (non-cardiomyocytes), so it is necessary to purify cardiomyocytes before further investigations. To date, several methods have been used for cardiomyocyte purification (4), including percoll gradient centrifugation (5), genetic modification [to generate expression of an identifier molecule driven by a cardiac-specific promoter (6, 7)], and mitochondrial dyes or antibodies against cardiac specific markers (8–11). However, these techniques all possess significant drawbacks, including low efficiency (percoll gradient centrifugation), safety concerns for therapeutic applications (genetic modification), and relatively high experimental cost (mitochondrial dyes and cardiac specific markers).

The heart’s continuous mechanical function necessitates a unique metabolic profile for its constituent cardiomyocytes, which could be exploited for isolation of these cells. The two primary metabolic substrates for cardiomyocytes are glucose and fatty acid. In glycolysis, glucose is converted to pyruvate and two ATP molecules. Pyruvate is then utilized in the tricarboxylic acid (TCA) cycle in mitochondria with the generation of an additional 36 ATP molecules. In anaerobic metabolism, lactate can be converted to pyruvate by lactate dehydrogenase, which can subsequently be utilized as the substrate in the TCA cycle. In the first report of cardiomyocyte purification by using a metabolic method, Fukuda and colleagues demonstrated that glucose-depleted culture medium supplemented with lactate could be used to purify mouse and human PSC-derived cardiomyocytes (12).

However, immature cardiomyocytes use glucose as a primary substrate for generation of ATP. On the other hand, fatty acid is essential for realizing the metabolic demands of mature cardiomyocytes and represents the substrate for more than 90% of energy production in the adult heart (13). This shift from glucose to fatty acid as a primary metabolic substrate represents a key event in cardiomyocyte maturation (14); it is unknown whether replacement of a glucose-rich environment with one replete with fatty acid could help to facilitate this change. Moreover, it is known that certain molecules including 3,3′,5-Triiodo-l-thyronine (T_3_), insulin-like growth factor 1 (IGF-1), and micro RNA miR-1 can promote cardiomyocyte maturation *in vitro* (14). Among these molecules, T_3_ is known to positively regulate cardiac genes including *MYH6, TNNI3, NKX2.5, SERCA*, and *RYR2* (14–16). More importantly, T_3_ can promote fatty acid oxidation (FAO) by upregulating several rate-limiting enzymes in FAO and mitochondrial biogenesis (17, 18), which may facilitate the metabolic switch from immature to mature cardiomyocytes.

Based on these data, we hypothesized that using fatty acid to replace glucose in the culture medium can both promote purification and enhance maturation of PSC-derived cardiomyocytes and that supplementation with T_3_ would potentiate this process. Indeed, we found that glucose-depleted culture medium supplemented with fatty acid and T_3_ can be used for purification of hPSC-derived cardiomyocytes. Moreover, compared to untreated control cells, treated cardiomyocytes exhibited a phenotype more consistent with mature cardiomyocytes, as evidenced by action potential (AP) characteristics, high sensitivity to isoproterenol, sarcomeric organization, proliferative activity, and expression levels of various ion channel and cardiac-specific genes. This highly efficient and low-cost method of hPSC-derived cardiomyocyte purification may be suitable for multiple applications where mature cardiomyocytes are required.

## Materials and Methods

### Cell Culture

Human pluripotent stem cells [WA07 (H7)] from WiCell Research Institute (WI, USA), NCRM1 iPSC line from Codex BioSolutions Inc. (MD, USA), and BJ-iPSCs derived from human fibroblast cells [CRL-2522, ATCC (VA, USA)] were plated on Geltrex LDEV-Free Reduced Growth Factor Basement Membrane Matrix (Gibco, A1413202)-coated plates, and then were cultured with Essential 8 Medium (Gibco, A1517001). Experimental results and figures in this paper were obtained mainly using hESCs (WA07) and confirmed by other hiPSCs. The differentiation protocol was modified based on the published protocols (1, 2). Briefly, hPSC were treated with small molecule CHIR99021 (Tocris, 4423, final concentration 10 μM) in the RPMI-BSA medium [RPMI 1640 Medium (HyClone, SH30027.01) supplemented with 213 μg/ml AA2P (l-ascorbic acid 2-phosphate magnesium) (A8960, Sigma) and 0.1% bovine serum albumin (BSA) (A1470, Sigma)] for 24 h, then were incubated with RPMI-BSA medium for 48 h. On differentiation day 4, cells were treated with the small molecule IWP2 (Tocris, 3533, final concentration 5 μM) in RPMI-BSA medium. After 48 h, media were changed to RPMI-BSA medium. Then, RPMI 1640 Medium supplemented with 3% KnockOut Serum Replacement (Gibco, 10828-028, the routine medium) was used to culture the cardiomyocytes in the following experiments. In general, contracting cardiomyocytes could be observed on differentiation day 9–11.

### Metabolic Selection

According to the previous report (12), lactate medium was prepared as DMEM Medium (No Glucose) (Gibco, 11966-025) supplemented with Sodium DL-lactate (Sigma, L4263, final concentration 4 mM). Fatty acid medium was prepared as DMEM Medium (No Glucose) supplemented with 0.1% BSA (Sigma, A1470) and 1× Linoleic Acid-Oleic Acid-Albumin (Sigma, L9655). Fatty acid + T_3_ medium was fatty acid medium supplemented with T_3_ (Acros Organics, 437260010, final concentration 10 nM). Cells were treated with metabolic selection medium (lactate, fatty acid and fatty acid + T_3_) for purification and cultured with routine medium as controls. The medium was changed every 2 days and the whole selection process lasted no longer than 9 days.

### Cell Viability Test

Human induced pluripotent stem (iPS) cells, human embryonic stem (ES) cells, mouse ES cells, mouse neonatal cardiomyocytes, and mouse HL-1 cells were exposed to metabolic selection medium (lactate and fatty acid) and glucose-free DMEM medium. At each time point, cells were trypsinized using 0.25% Trypsin-EDTA (Gibco, 25200-056). After serum neutralization, the trypsinized cells were centrifuged for 4 min at 1,000 rpm, resuspended in 100 µl phosphate-buffered saline (PBS), stained with 0.4% Trypan Blue Solution (Gibco, 15250-061), and counted using a hemocytometer. The cell viability rate equals the number of live cells/the cell number at the beginning of purification.

### Intracellular Staining for Fluorescence-Activated Cell Sorting (FACS) Using Troponin T Cardiac Isoform Antibody

Cardiomyocytes were dissociated using 0.05% Trypsin-EDTA and then fixed with 4% paraformaldehyde (Electron Microscopy Sciences, 15714-S) for 20 min at room temperature. Cells were permeabilized and blocked with 1× PBS supplemented with 0.15% Triton X-100 (Sigma, T9284) and 10% goat serum (Millipore, S26-LITER) for 15 min at room temperature. Then cells were washed twice with 1× PBS supplemented with 0.2% Tween 20 (Bio-Rad, 1706531) and 0.1% BSA. Subsequently, cells were stained with Troponin T Cardiac Isoform antibody (Thermo Fisher, MA5-12960) at room temperature for 1 h. After washing with 1× PBS containing 0.2% Tween 20 and 0.1% BSA, cells were incubated with the Alexa Fluor 488 goat anti-mouse IgG secondary antibody (Thermo Fisher, A-11001) at room temperature for 1 h. These cells were sorted using the LSRII analyzer (BD). FACS results were analyzed using FlowJo software.

### Immunofluorescence Staining

Cardiomyocytes were dissociated using 0.05% Trypsin-EDTA and plated onto microscope cover slips (Fisher Scientific, 051115-9) prior to purification by metabolic selection medium. After purification, cells were fixed with 4% paraformaldehyde at room temperature for 20 min and washed three times with 1× PBS. Cells were then permeabilized with PBS containing 0.25% Triton X-100 at room temperature for 10 min. After incubating in the blocking buffer (1× PBS with 10% goat serum), cells were stained with different primary antibodies at 4°C overnight (Troponin T Cardiac Isoform antibody, Thermo Fisher, MA5-12960; Cardiac Troponin I antibody, Abcam, ab47003; Anti-α-Actinin (Sarcomeric) antibody, Sigma, A7811; Anti-IK1 antibody, Santa Cruz, sc-365265; Anti-SERCA2 antibody, Santa Cruz, sc-73022; Anti-CPTI antibody, Santa Cruz, sc-393070). Cells were washed three times with PBS containing 0.1% Triton X-100, then incubated with the Alexa Fluor 488 goat anti-mouse or Alexa Fluor 555 goat anti-rabbit IgG secondary antibodies at room temperature for 1 h. Nuclei were labeled with DAPI (4′,6-diamidino-2-phenylindole, 1 μg/ml) for 5 min. Cells were observed using AxioObserver Inverted SK-2 microscopy (Zeiss). Image analysis was performed with ImageJ software. Quantification of the FITC fluorescence intensity for each condition was performed using Zen 2.3 lite software (Zeiss). For measurement of sarcomere length, we followed the protocol from the previous report (14). Briefly, myofibrils with at least five continuous and organized α-Actinin-positive bands were selected and measured. Length measurement was performed using Zen 2.3 lite software (Zeiss).

### AP Recording

All electrophysiological recordings were conducted at room temperature using an Axonmulticlamp 700B Amplifier and a pClamp system (versions 10.2, Axon Instruments). For spontaneous AP current clamp recordings, pipettes were filled with a solution containing (in mmol/l): KCl 135, MgCl_2_ 1, EGTA 10, HEPES 10, and glucose 5, pH 7.2 with KOH. The bath solution contained (in mmol/l): NaCl 136, KCl 4, CaCl_2_ 1, MgCl_2_ 2, HEPES 10, and glucose 10, pH 7.4 with NaOH. The AP maximum upstroke velocity (dV/dt_max_), the maximum negative potentials, action potential amplitudes (APAs), as well as AP durations at 50% (APD_50_) and 90% (APD_90_) of repolarization were measured. To avoid the influences of the spontaneous beating rates on the APD, the corrected APD (cAPD) by heart rates [APD/square root of the cycle length between two spontaneous APs (RR)] was used for average and compared between different groups (19).

### Calcium Imaging

Intracellular Ca^2+^ transients were studied by using the IonOptix microfluorimetry system (IonOptix Inc., MA, USA). According to the standard protocol, cardiomyocytes derived from hPSCs were loaded with Fluo-8/AM (Molecular Probes) in Tyrode buffer for 60 min at 37°C. Then, cardiomyocytes were perfused with Tyrode buffer supplemented with 1.8 mM CaCl_2_, 5.5 mM d-glucose, and 0.5 m Mprobenecid and maintained at 35°C–37°C. Cells were field stimulated at 0.5 Hz using a MyoPacer Field Stimulator (IonOptix Inc., MA, USA). Intracellular calcium transients were analyzed with IonWizard software. The Ca^2+^ transient peak amplitude was denoted *F*/*F*_0_, in which *F*_0_ presented the fluorescence intensity at the onset of the experiment. SR calcium content of store was evaluated following rapid caffeine application (10 mM). To evaluate the effect of isoproterenol administration, intracellular Ca^2+^ transients were measured before and after isoproterenol (100 nM) application.

### Quantitative PCR

Total RNA was extracted using the Qiagen RNeasy Mini Kit (Qiagen, 74104) prior to the treatment with DNase I (Thermo Fisher, EN0525) for 30 min. mRNA was reverse transcribed using iScript Reverse Transcription Supermix (Bio-Rad, 1708841). Quantitative PCR was performed using a Mastercycler RealPlex^2^ (Eppendorf) with SsoFast EvaGreen Supermix (Bio-Rad, 1725200). The quantitative PCR primers designed from qPrimerDepot (20) are as followed (from 5′ to 3′):

**Table:** 

*KCNA4*-Forward:	CAGCCAAAATCATGCAGAAG;
*KCNA4*-Reverse:	GATCATTCTTCCCCTCCTCC;
*KCND3*-Forward:	AAACAATCACAGGGACTGGC;
*KCND3*-Reverse:	ACACCATTGTCACCATGACC;
*KCNH2*-Forward:	TCCTTCTCCATCACCACCTC;
*KCNH2*-Reverse:	AAATCGCCTTCTACCGGAAA;
*KCNQ1*-Forward:	ACAAAGTACTGCATGCGTCG;
*KCNQ1*-Reverse:	CATGAGAACCAACAGCTTCG;
*KCNN4*-Forward:	GGACCTCTTTGGCATGAAAG;
*KCNN4*-Reverse:	CATGCAGAGATGCTGTGGTT;
*CACNA1C*-Forward:	TAGGCATTGGGGTGAAAGAG;
*CACNA1C*-Reverse:	GAAGATGATTCCAACGCCAC;
*SCN5A*-Forward:	GAGGGCAATGATCTTGAAGG;
*SCN5A*-Reverse:	TCAACACACTCTTCATGGCG;
*TNNI3*-Forward:	GCCCACCTCAAGCAGGTG;
*TNNI3*-Reverse:	TTGCGCCAGTCTCCCACCTC;
*TNNI1*-Forward:	ACAAGGTGCTGTCTCACTGC;
*TNNI1*-Reverse:	CTCTTCAGCAAGAGTTTGCG;
*MYH6*-Forward:	CAAGTTGGAAGACGAGTGCT;
*MYH6*-Reverse:	ATGGGCCTCTTGTAGAGCTT;
*MYH7*-Forward:	GGGCAACAGGAAAGTTGGC;
*MYH7*-Reverse:	ACGGTGGTCTCTCCTTGGG;
*CPT1A*-Forward:	GCCTCGTATGTGAGGCAAA;
*CPT1A*-Reverse:	CCCATTCGTAGCCTTTGGTA;
*CPT1B*-Forward:	GCTTGATTTCTTCACGGTCC;
*CPT1B*-Reverse:	CCTCTCATGGTGAACAGCAA;
*ACOX1*-Forward:	ATGCCCAAGTGAAGATCCAG;
*ACOX1*-Reverse:	GAGGTGGCTGTCAGGAAAAG;
*PPARGC1A*-Forward:	CTGCTAGCAAGTTTGCCTCA;
*PPARGC1A*-Reverse:	GCTTTCTGGGTGGACTCAAG;
*NRF1*-Forward:	GCCACTGCATGTGCTTCTAT;
*NRF1*-Reverse:	GTCGCAGTCTCCACGGC.

### BrdU Assay

The BrdU assay was performed using a Bromo-2′-deoxy-uridine Labeling and Detection Kit I (Roche, 11296736001). We followed the instructions provided by the manufacturer prior to double-staining with cardiac Troponin I antibody (Abcam, ab47003). Cells were incubated with the primary antibody for 1 h at 37°C, then washed three times with PBS containing 0.1% Triton X-100. Cells were incubated with the Alexa Fluor 488 goat anti-rabbit IgG secondary antibody and the Alexa Fluor 555 goat anti-mouse IgG secondary antibody at room temperature for 1 h. Images were captured using AxioObserver Inverted SK-2 microscopy (Zeiss) and analyzed using ImageJ software.

### Statistics

Values are expressed as mean ± SD. Statistical significances were evaluated using one-way ANOVA with Bonferroni correction. *P* < 0.05 was considered statistically significant. The statistical significances between experimental groups in each figure were shown in Table S1 in Supplementary Material.

## Results

**Figure 1:**
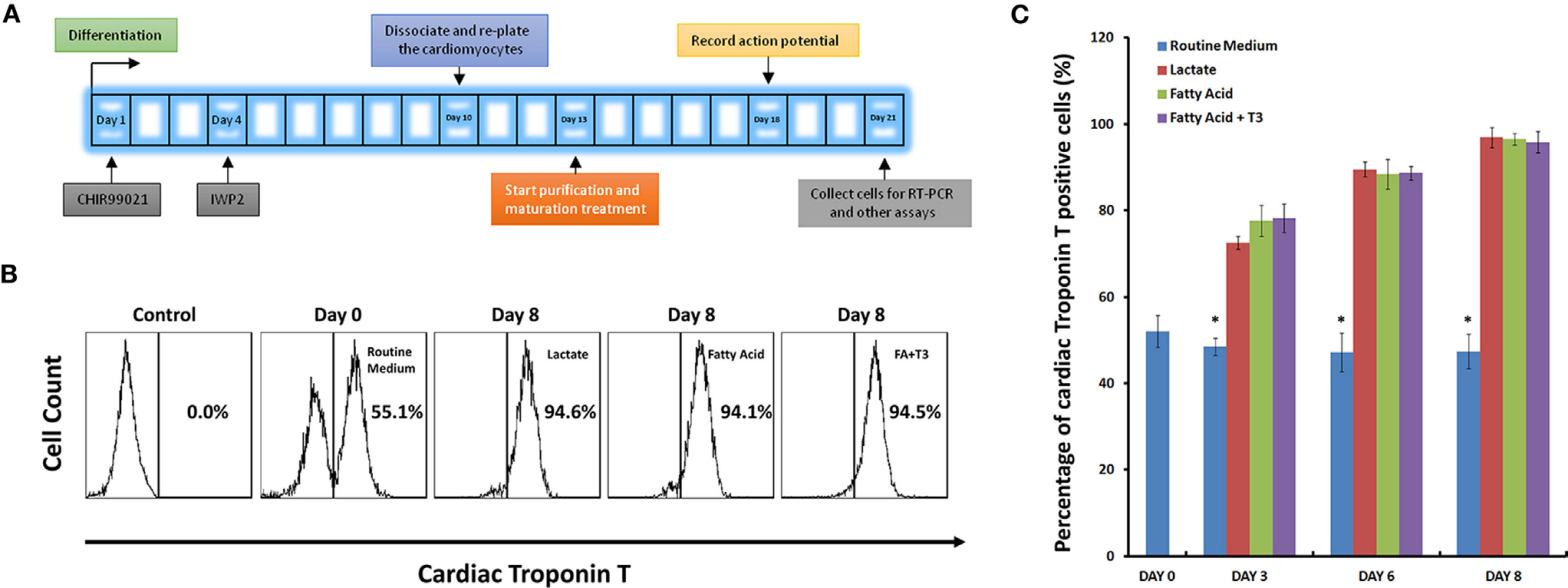
High purity of cardiomyocytes can be obtained under glucose-depleted and fatty acid (with or without T_3_)-supplemented conditions. **(A)** The schematic for purification and maturation using metabolic selection. Glycogen synthase kinase 3 inhibitor CHIR99021 and Wnt pathway inhibitor IWP2 (gray boxes) were added to the differentiation medium on differentiation days 1 and 4, respectively. On differentiation day 10, cells were dissociated and plated on glass cover slips. After 3 days recovery, cells were cultured with metabolic selection medium (No-glucose DMEM medium supplemented with lactate, fatty acid, and fatty acid + T_3_). On differentiation day 18, cells were collected for patch clamping experiments. On differentiation day 21, cells were collected for other experiments. **(B)** Representative fluorescence-activated cell sorting (FACS) analyses of cardiac Troponin T expression in the human pluripotent stem cell (hPSC)-derived cells on day 0 with routine culture medium, and on day 8 with metabolic selection medium. In the control group, the isotype control (mouse IgG) was used instead of the primary antibody. **(C)** Time courses of selection efficiency using the lactate-supplemented condition (red), the fatty acid-supplemented condition (green), and the fatty acid with T_3_-supplemented condition (purple); cells cultured with routine medium (blue) were set up as the control groups (*n* = 3). The asterisk means each test group is significantly different from controls, *P* < 0.01.

A protocol for human iPS cell differentiation to a cardiac lineage was modified from the method previously described, allowing acquisition of contracting cardiomyocytes as early as on differentiation day 9 (1, 2). Differentiated cells would then be subjected to an 8-day metabolic selection process. Figure 1A represents a schematic of this purification and maturation treatment.

### Glucose-Depleted Culture Medium Supplemented with Fatty Acid Can Enrich for hPSC-Derived Cardiomyocytes

First, we assessed the relative abilities of multiple cell lines to tolerate various culture conditions. Human iPS cells, human ES cells, murine ES cells, murine neonatal cardiomyocytes, and murine HL-1 cells were cultured in non-glucose medium, non-glucose medium supplemented with lactate, and non-glucose medium supplemented with fatty acid (Figure S1 in Supplementary Material). With the exception of murine neonatal cardiomyocytes, no cell type could survive without glucose beyond 5 days. Culturing in lactate—compared to fatty acid-supplemented non-glucose media exerted similar effects on human iPS cells, human ES cells, murine ES cells, and murine neonatal cardiomyocytes. Interestingly, murine HL-1 cells appeared more adaptive to the lactate-supplemented glucose-free medium, likely as a consequence of the general preference of neoplastic cells for lactate as a metabolic substrate (21). These data suggested a relative selection bias for cardiomyocytes as compared to other cell types when cultured in glucose-free conditions supplemented with either lactate or fatty acid.

Next, we compared the purification efficiency of several methods of metabolic selection. hPSC-derived cardiomyocytes were cultured in glucose-free medium supplemented with lactate, fatty acid, or a combination of fatty acid and T_3_. After 8 days in these conditions, cells were dissociated, stained with anti-cardiac Troponin T (*TNNT2*) antibody, and analyzed by FACS. As defined by positivity for *TNNT2*, each of these three media conditions demonstrated similar purification efficiency of hPSC-derived cardiomyocytes (up to 95%, Figures 1B,C; Figure S2 in Supplementary Material).

### Fatty Acid and T_3_-Treated Cardiomyocytes Exhibited Mature AP Morphology

**Figure 2:**
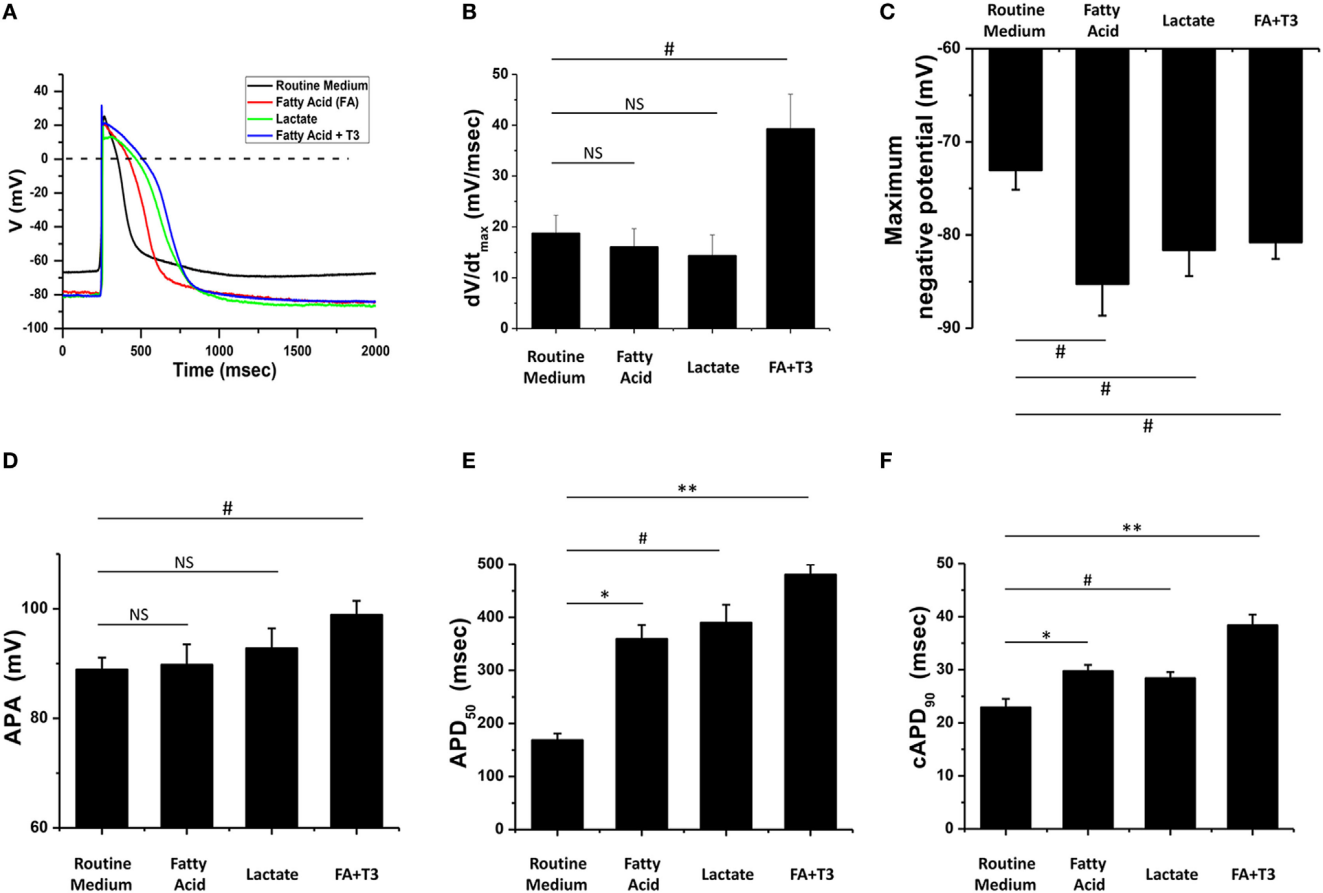
Fatty acid and T_3_-treated cardiomyocytes exhibited more mature action potential (AP) morphology. **(A)** Representative AP traces of cardiomyocytes cultured with metabolic selection medium and routine medium. **(B–F)** AP indices of dV/dt_max_, the maximum negative potential, action potential amplitude (APA), AP duration at 50% of repolarization (APD_50_), and the corrected AP duration at 90% of repolarization (cAPD_90_) from cardiomyocytes cultured with metabolic selection medium and routine medium (routine medium, *n* = 20 cells; fatty acid, *n* = 18 cells; lactate, *n* = 17 cells; fatty acid + T_3_, *n* = 20 cells). Only the combination of fatty acid + T_3_ treatment significantly improved all indices. NS, no significant difference; ^#^*P* < 0.05; *P < 0.01; **P < 0.001.

Action potential morphology is one of the basic indices of electrophysiological maturity of cardiomyocytes, while mature hPSC-derived cardiomyocytes should demonstrate similar AP morphology when compared to adult cardiomyocytes (14, 16). We cultured hPSC-derived cardiomyocytes in glucose-containing medium (Routine Medium), as well as several glucose-depleted conditions [medium supplemented with lactate (Lactate), medium supplemented with fatty acid (Fatty Acid), and medium supplemented with fatty acid and T_3_ (Fatty acid + T_3_)]. After culturing for 5 days in these conditions, AP morphology was assessed with whole-cell patch clamping. Compared to cardiomyocytes cultured in the routine medium, fatty acid and T_3_-treated cardiomyocytes exhibited AP morphology more consistent with adult cardiomyocytes (Figure 2). Notably, the combination of fatty acid and T_3_ was the only culture condition that could improve all the characteristics of AP, including dV/dt_max_, the maximum negative potential, APA, AP duration at 50% of repolarization (APD_50_), and the corrected AP duration at 90% of repolarization (cAPD_90_). Likewise, compared to the routine culture condition, culturing in lactate or fatty acid conditions could also make the maximum negative potential more hyperpolarized and prolong the AP duration, but had no significant (NS) effect on dV/dt_max_ or APA.

### Fatty Acid and T_3_-Treated Cardiomyocytes Demonstrated Improved Response to Caffeine and Isoproterenol

**Figure 3:**
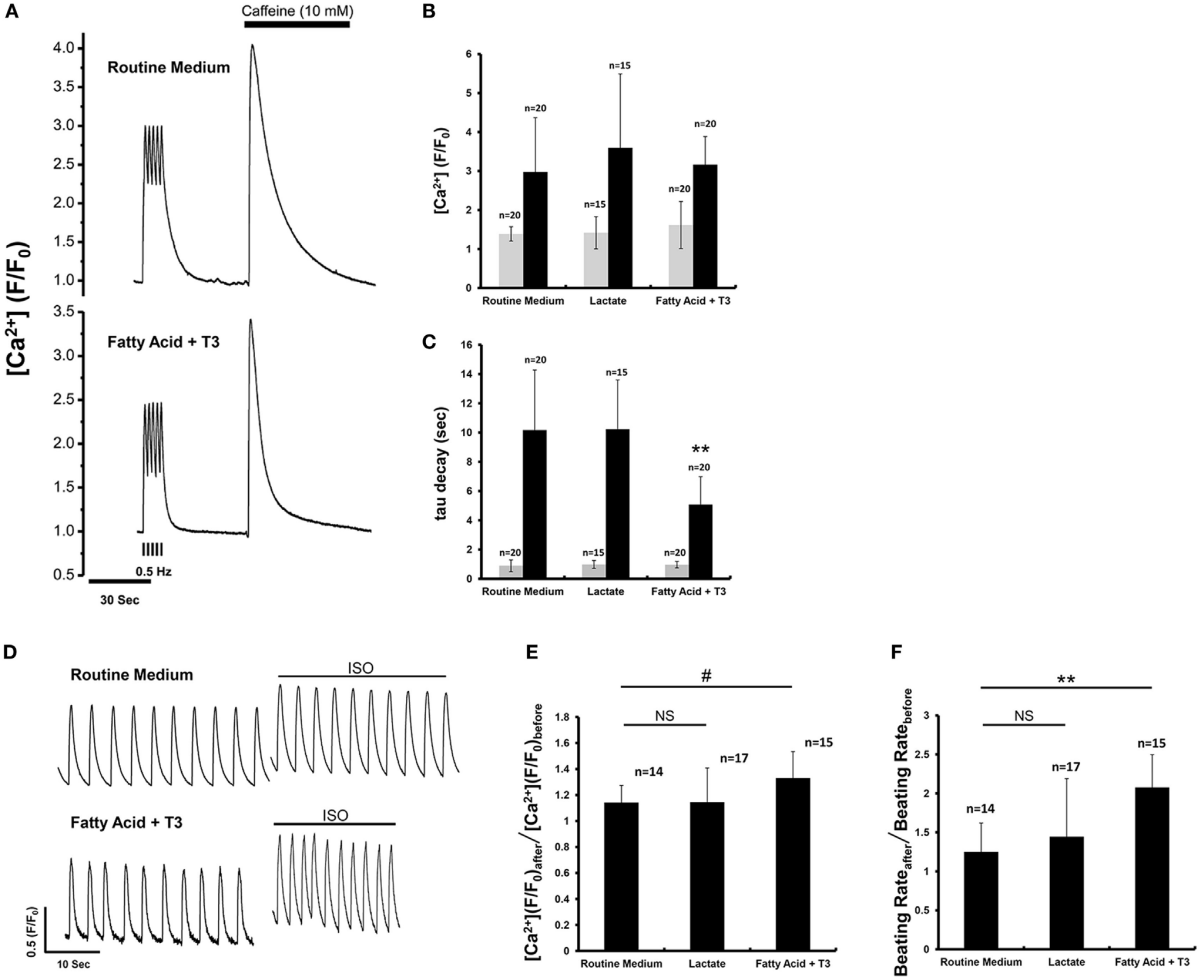
Fatty acid and T_3_-treated cardiomyocytes demonstrated improved calcium-handling in response to caffeine and isoproterenol. **(A)** The representative traces of calcium transient before and after caffeine application. **(B,C)** Calcium transients peak amplitude and decay time constant (tau) before (gray boxes) and after (black boxes) caffeine administration from cardiomyocytes maintained in the various culture conditions. Before caffeine administration, calcium transient peak amplitude and tau were recorded from the 0.5 Hz-paced cardiomyocytes. The double asterisks mean tau values of fatty acid and T_3_-treated cardiomyocytes were significantly different from the values of other two experimental groups. **(D)** The representative traces of calcium transient before and after isoproterenol application. **(E)** The ratio of calcium transient peak amplitude after isoproterenol application to that before isoproterenol application. Fatty acid and T_3_-treated cardiomyocytes demonstrated a greater response to isoproterenol than the other groups. **(F)** The ratio of beating rates after isoproterenol application to that before isoproterenol application. *n* indicates tested cells. ^#^*P* < 0.05; **P < 0.001.

We performed calcium imaging with a microfluorimetry system to assess effects of the three culture conditions (routine medium, lactate, and fatty acid + T_3_) on calcium handling. There were NS differences for the Ca^2+^ transient peak amplitude and decay time constant between different culture conditions while pacing at 0.5 Hz (Figures 3A–C). Caffeine was then administered to evaluate the sarcoplasmic reticulum Ca^2+^ content of the store (22). There were NS differences for the Ca^2+^ transient peak amplitude after caffeine application. However, the tau of fatty acid and T_3_-treated cardiomyocytes was significantly reduced compared to cardiomyocytes from the other culture conditions (Figure 3C), which was suggestive that fatty acid and T_3_ treatment could improve the function of sodium–calcium exchanger. Isoproterenol was administered to evaluate the effects of β_1_ and β_2_ agonism. After isoproterenol application, the peak amplitude of Ca^2+^ transients and beating rates were increased in cardiomyocytes from each of the three culture conditions. However, the response to isoproterenol was significantly greater in fatty acid and T_3_-treated cardiomyocytes (Figures 3D–F).

### Fatty Acid and T_3_ Treatment Associated With Increased Expression of Major Ion Channel and Cardiac-Specific Genes

**Figure 4:**
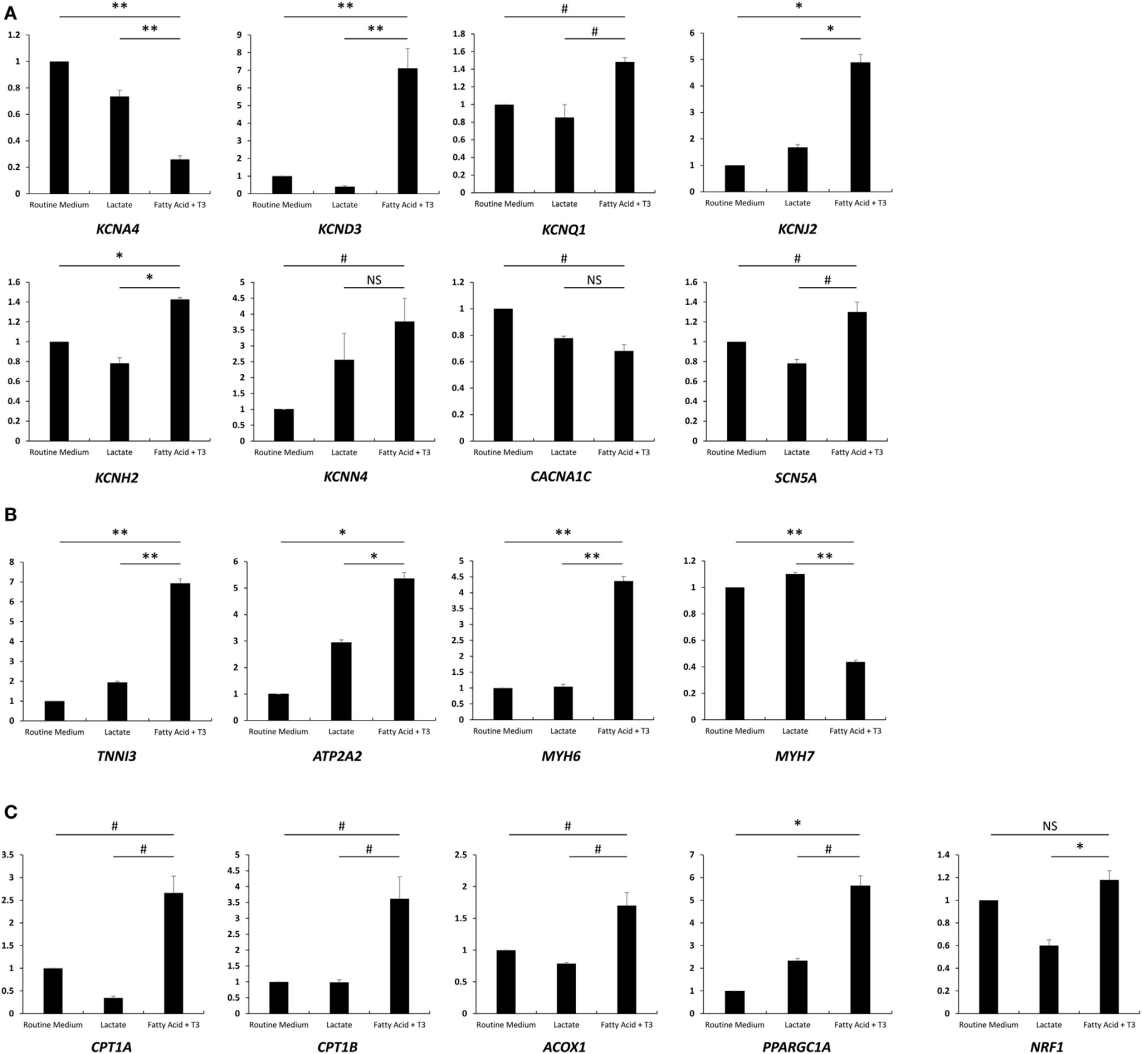
Fatty acid and T_3_ treatment is associated with increased expression of major ion channel and cardiac-specific genes. Total mRNA was extracted from cells cultured in the lactate-supplemented condition, the fatty acid + T_3_-supplemented condition, as well as routine medium (*n* = 3). Expression of major ion channel genes, cardiac genes, and fatty acid oxidation-related genes are shown in **(A–C)**, respectively. NS, no significant difference; ^#^*P* < 0.05; *P < 0.01; **P < 0.001.

As a hormone, T_3_ binds nuclear receptors and regulates the transcription of various genes (23, 24). We measured the expression of major ion channel and cardiac genes in each of the three culture conditions compared to the routine medium condition. We noted significant upregulation of genes encoding for the major cardiac potassium channel subunits associated with the fatty acid and T_3_ treatment: *KCND3* and *KCNQ1* expression, which are known to be more pronounced in canine adult (as compared to neonatal) ventricular tissue (25), were increased 7.1- and 1.5-fold, respectively. *KCNJ2*, which encodes for the pore of the inward rectifier K^+^ channel (26), a key regulator of phase 3 and known to be upregulated in cardiac development (27), increased 4.9-fold. *KCNH2* and *KCNN4* increased 1.4- and 3.8-fold, respectively. The exception was *KCNA4*, which appeared to be downregulated (Figure 4A). *SCN5A*, which encodes for Nav1.5 and is responsible for phase 0 of the cardiac AP, was upregulated 1.3-fold with culture in fatty acid and T_3_, while the expression of *CACNA1C* (encoding for Cav1.2 of the voltage dependent l-type calcium channel) was downregulated (Figure 4A). Overall, these results are consistent with previously published data in murine, rat, and canine cardiomyocytes and suggest the emergence of a more mature phenotype associated with fatty acid and T_3_ treatment.

We next examined relative expression levels of cardiac-specific genes (Figure 4B). We found that the expression of *TNNI3*, encoding for the cardiac biomarker troponin I (14), was significantly increased after fatty acid + T_3_ treatment (6.93-fold versus culture in routine medium). This is consistent with data suggesting that T_3_ can increase expression of *TNNI3* in the rat heart (28). *ATP2A2* is also known to be upregulated with cardiomyocyte maturation (14); its product, SERCA2, plays a pivotal role in excitation-contraction coupling in mature cardiomyocytes (29). Unsurprisingly, *ATP2A2* exhibited a 5.4-fold increase in expression when cardiomyocytes were cultured in fatty acid and T_3_ compared to cardiomyocytes in the routine culture medium, which is suggestive of improved calcium-handling and consistent with the microfluorimetry data. Interestingly, *MYH6* (encoding for the α isoform of the myosin heavy chain) was upregulated and *MYH7* (encoding for the β isoform of myosin heavy chain) was downregulated, which is discrepant with protein levels in human hearts, where the β isoform predominates over the α isoform (30, 31). However, our results were similar to the previous reports (14, 32). This might be one of the transcriptional regulation effects of T_3_. Unsurprisingly, the genes encoding for carnitine palmitoyltransferase I and Acyl-CoA oxidase (*CPT1A, CPT1B*, and *ACOX1*), the rate-limiting enzymes for FAO, were all upregulated with culture in fatty acid + T_3_ (Figure 4C). *PPARGC1A*, which encodes the protein involved in mitochondrial biogenesis, demonstrated enhanced expression after fatty acid + T_3_ treatment as well.

To confirm the results from quantitative real-time PCR (qPCR) assays, IK1, SERCA2, and CPTI were selected to perform immunostaining in cardiomyocytes from each condition. The microscopic observations were consistent with the results from qPCR (Figures S3–S5 in Supplementary Material).

### Fatty Acid and T_3_ Enhance Sarcomere Formation and Decrease Proliferative Activity in hPSC-Derived Cardiomyocytes

**Figure 5:**
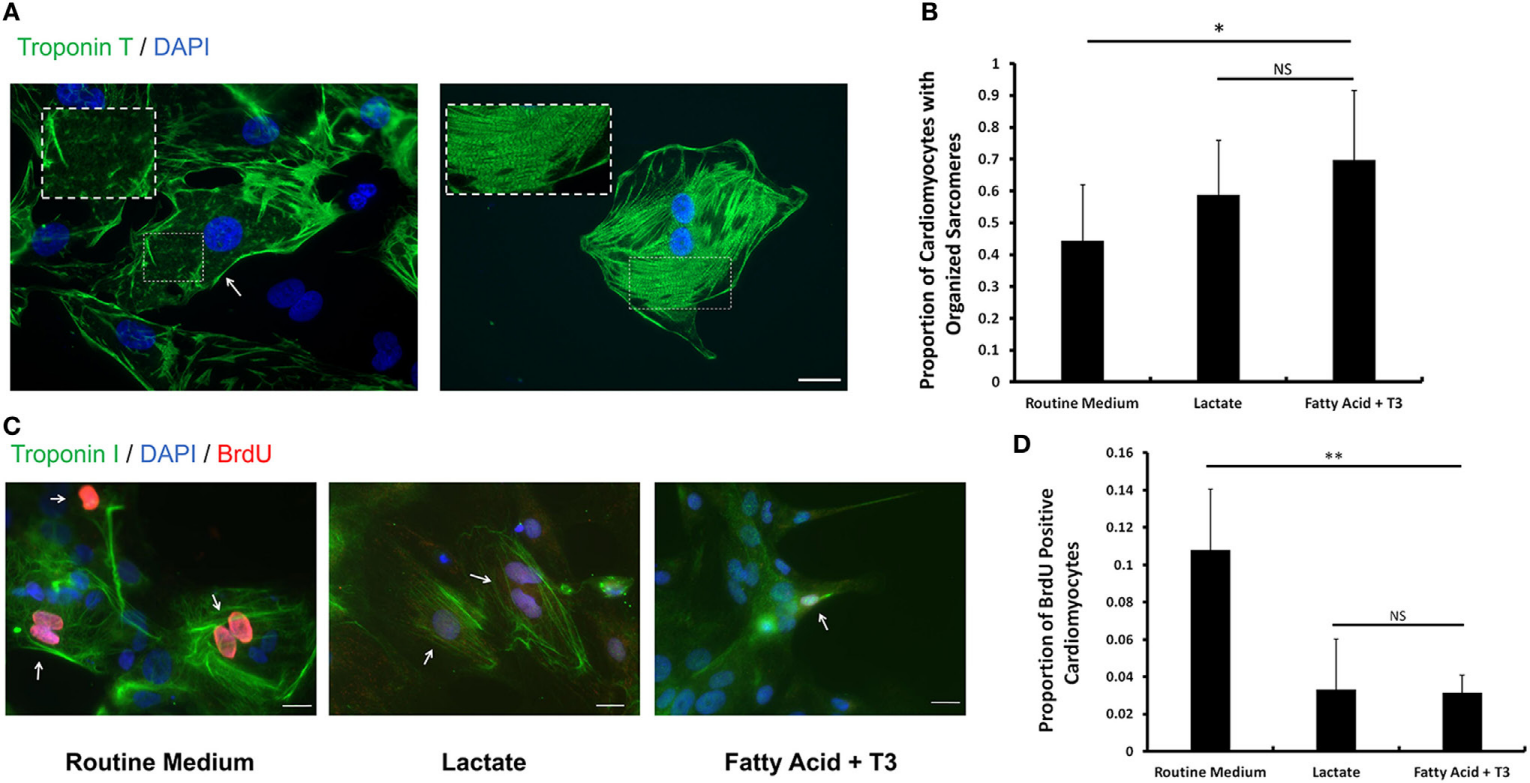
Fatty acid and T_3_ treatment enhances sarcomere formation and decreases proliferative activity in human pluripotent stem cell (hPSC)-derived cardiomyocytes. **(A)** Representative photomicrographs showing cardiac Troponin T (green) in hPSC-derived cardiomyocytes with unorganized (left panel) and organized (right panel) sarcomeres. Magnification of the sarcomeres is shown in the white dash line boxes. Nuclei are stained with DAPI (blue). The white arrow indicates the cardiomyocyte with undeveloped sarcomeres cultured in the routine medium. The scale bar is 200 µm. **(B)** The proportion of cardiomyocytes with organized sarcomeres from the lactate-supplemented condition, the fatty acid + T_3_-supplemented condition and the routine culture condition. *n* > 1300 cells in each group. ^#^*P* < 0.05; *P < 0.01. **(C)** Fatty acid and T_3_-treated cardiomyocytes demonstrate lower proliferative activity. Bromo-2′-deoxy-uridine labeling was performed. Cells were co-stained with cardiac Troponin I antibody (green), DAPI (blue), and BrdU antibody (red). Representative images were taken from cells cultured in control medium, in fatty acid + T_3_ medium, and in lactate medium. The white arrows denote BrdU-positive cardiomyocyte nuclei. The scale bar is 20 µm. **(D)** Fatty acid + T_3_ treatment and lactate treatment are associated with a significantly lower percentage of BrdU-positive cardiomyocytes than the control condition. Approximately 8,000 cells were counted in each group. NS, no significant difference; **P < 0.001.

The sarcomeric structure is one of the hallmarks of mature cardiomyocytes (14). Following the criteria from previous reports (33, 34), we estimated the proportion of cardiomyocytes with organized sarcomeres in cells cultured in each of the three conditions. We found that a higher proportion of fatty acid and T_3_-treated cardiomyocytes displayed organized sarcomeres compared to the routine medium condition (Figures 5A,B). The sarcomere length of cardiomyocytes from each culture condition was also measured. Fatty acid and T_3_-treated cardiomyocytes exhibited increased sarcomere length compared to controls (Figure S6 in Supplementary Material). It has been reported that exposure to T_3_ can decrease the proliferation rate of iPSC-derived cardiomyocytes (14). Then, we utilized a BrdU-based cell proliferation assay to evaluate hPSC-derived cardiomyocytes cultured in each of the three conditions. We demonstrated that the proportion of BrdU positive cardiomyocytes was significantly decreased in both fatty acid + T_3_ (0.032 ± 0.009 versus 0.108 ± 0.032 in control cells, *P* < 0.001) and lactate (0.033 ± 0.027 versus 0.108 ± 0.032 in control cells, *P* < 0.001) culture conditions (Figures 5C,D). This is suggestive that metabolic selection with fatty acid + T_3_ in hPSC-derived cardiomyocytes may improve the development of sarcomeric structure and lead to lower proliferative activity of cardiomyocytes, which were observed in adult cardiomyocytes.

## Discussion

Applications for hPSCs are rapidly expanding, especially in the realm of cardiovascular research. hPSC-derived cardiomyocytes are used to probe mechanisms of cardiovascular pathology, assess drug safety, and be a possible therapeutic tool. This last use is regarded by many as the “holy grail” of cardiovascular therapeutics. As the most frequent cause of death in the developed world, ischemic heart disease is incited by an initial loss of cardiomyocytes during acute myocardial infarction, which serves subsequently as an impetus for myocardial remodeling. The ability to replace lost or injured cardiomyocytes with mechanically and electrically healthy counterparts, therefore, represents an effective treatment for many forms of cardiovascular diseases: hPSCs represent an unlimited source for such healthy cardiomyocytes.

There are several major hurdles that must first be overcome before the myriad potential uses of hPSC-derived cardiomyocytes can be realized. One of these is the purification of generated cardiomyocytes. Despite recent advancements in the efficiency of cardiac differentiation of hPSCs, the resulting cell population remains a heterogeneous pool of cardiomyocytes and other cells (35). Since cardiomyocytes exhibit marked phenotypic differences from other cell-types, the validity of conclusions drawn from their investigational use and the safety of their therapeutic use is predicated upon their purification from non-cardiomyocytes.

According to the unique metabolic features of cardiomyocytes, it is logical to make an assumption that cardiomyocytes, but not non-cardiomyocytes, are able to survive by taking advantage of certain metabolic substrates under metabolic selection. Fukuda and his colleagues first reported that PSC-derived cardiomyocytes could be purified by a glucose-depleted medium supplemented with lactate. Their solid work clarified that metabolic selection was a high-efficiency way for the enrichment of cardiomyocytes. In their research, the distinct metabolic differences between cardiomyocytes and ES cells in transcriptome were fully demonstrated. Genes involved in the TCA cycle exhibited higher expression levels in cardiomyocytes than in ES cells. They also proved that cardiomyocytes could use lactate as an alternative energy substrate more efficiently than non-cardiomyocytes, so only cardiomyocytes could obtain essential ATPs and survive in the glucose-depleted culture medium supplemented with lactate. As we know, glucose and fatty acid are two major metabolic substrates for TCA cycle. Glucose can be converted into pyruvate then pyruvate turns into Acetyl CoA; fatty acid can also be converted into Acetyl CoA through several steps. In our study, fatty acid, an alternative metabolic substrate of cardiomyocytes, was selected as a replacement of glucose in the purification culture medium. Our data clearly illustrated that glucose-depleted medium supplemented with fatty acid could efficiently enrich hPSC-derived cardiomyocytes. Therefore, it is reasonable to deduce the mechanism of purification effect by using the glucose-depleted culture medium supplemented with fatty acid: like lactate, fatty acid can be used by cardiomyocytes with high efficiency to produce enough ATPs, while non-cardiomyocytes (especially stem cells) could not utilize fatty acid efficiently. So only cardiomyocytes could survive under this condition. More importantly, most hPSC-derived cardiomyocytes could survive under 8-day metabolic selections (the survival rates of cardiomyocytes under lactate or fatty acid + T_3_ conditions were as high as 96%, data not shown). It is important to note that free fatty acid has been reported as being toxic to cardiomyocytes (36, 37). This property can be ameliorated by supplementing the culture medium with BSA, which is reported to bind the free fatty acid, allowing for a complex that can be transported to the intracellular space (38, 39). For this reason, we supplemented our selection medium with BSA; we attribute the survival of cardiomyocytes in our study to this medium change. With this in mind, purification by metabolic selection should be carefully adopted for hPSC-derived cardiomyocytes from patients with endocrine or metabolic disorders, because these cardiomyocytes probably could not suffer from the metabolic stress, which leads to failure of cardiomyocyte enrichment.

Human pluripotent stem cell-derived cardiomyocytes spontaneously depolarize and contract. This feature is conferred by a prominent I_f_ and suggestive of an immature electrical phenotype. In fact, previous studies suggest that hPSC-derived cardiomyocytes more closely resemble fetal cardiomyocytes as compared to their adult counterparts (40). Despite the relative ability of hPSC-derived cardiomyocytes to detect delayed repolarization elicited by drugs with known effects on hERG or the clinical ECG (41–50), mature hPSC-derived cardiomyocytes are more ideal for probing mechanisms of adult-onset cardiovascular disease and for studies of pharmacological agents to be utilized in an adult population. As the shift from glucose to fatty acid as a primary metabolic substrate represents a key event in cardiomyocyte maturation (14), we hypothesized that this intervention could facilitate maturation. Moreover, since T_3_ can promote cardiomyocyte maturation (14), positively regulate cardiac genes (*MYH6, TNNI3, NKX2.5, SERCA*, and *RYR2*) (14–16) and upregulate several rate-limiting enzymes in FAO and mitochondrial biogenesis (17, 18), we hypothesized that this hormone could potentiate the maturation process. Indeed, compared to untreated control cells, treated cardiomyocytes exhibited a phenotype more consistent with mature cardiomyocytes, as evidenced by AP characteristics, sensitivity to isoproterenol, sarcomeric organization, proliferative activity, and expression levels of various ion channel and cardiac-specific genes.

dV/dt_max_, which reflects the rate of depolarization, is associated with the physiological and functional performance of *I*_Na_ (51). T_3_ might play a critical role in the enhancement of depolarization in cardiomyocytes. In addition to T_3_, we tested several other compounds with the reported ability to promote cardiomyocyte maturation (including phenylephrine, IGF-1, and WY-14643) in our culture system (52, 53). We found that only the combination of fatty acid and T_3_ could significantly improve the AP morphology of hPSC-derived cardiomyocytes, while other chemicals exhibited no differences compared to control culture conditions (data not shown).

In conclusion, we have developed an efficient and inexpensive method of purification and promoting maturation of hPSC-derived cardiomyocytes using glucose-depleted culture medium supplemented with fatty acid and T_3_. This method may be suitable for multiple applications where mature cardiomyocytes are required.

## Author Contributions

BL, XL, MS, and LB had substantial contributions to the design of the paper; BL, XL, MS, EW, YL, and LB contributed to the conception of the experiments and analyses of data; JL, XS and MD provided critical suggestions to improve the paper; BL, XL, MS, JL, and LB wrote the manuscript. All authors (BL, XL, MS, EW, YL, JL, XS, MD and LB) have read and approved the final manuscript.

## Conflict of Interest Statement

The authors declare that the research was conducted in the absence of any commercial or financial relationships that could be construed as a potential conflict of interest.
