# Supplementary material for: Culture in Glucose-Depleted Medium Supplemented with Fatty Acid and 3,3′,5-Triiodo-l-Thyronine Facilitates Purification and Maturation of Human Pluripotent Stem Cell-Derived Cardiomyocytes
Source: Front Endocrinol (Lausanne). 2017 Oct 9;8:253. doi: 10.3389/fendo.2017.00253 (PMC5641374; doi:10.3389/fendo.2017.00253)
Supplement: Supplementary file 1 [file Data_Sheet_1.PDF]

Supplementary Material

Culture in glucose-depleted medium supplemented with fatty acid and 3,3',5-Triiodo-L-thyronine facilitates purification and maturation of hPSC-derived cardiomyocytes

Bin Lin[1#], Xianming Lin[1#], Maxine Stachel[1], Elisha Wang[1], Yumei Luo[1,2], Joshua Lader[1], Mario Delmar[1] and Lei Bu[1*]

***Correspondence:**

Dr. Lei Bu

lei.bu@med.nyu.edu

Supplementary Figure

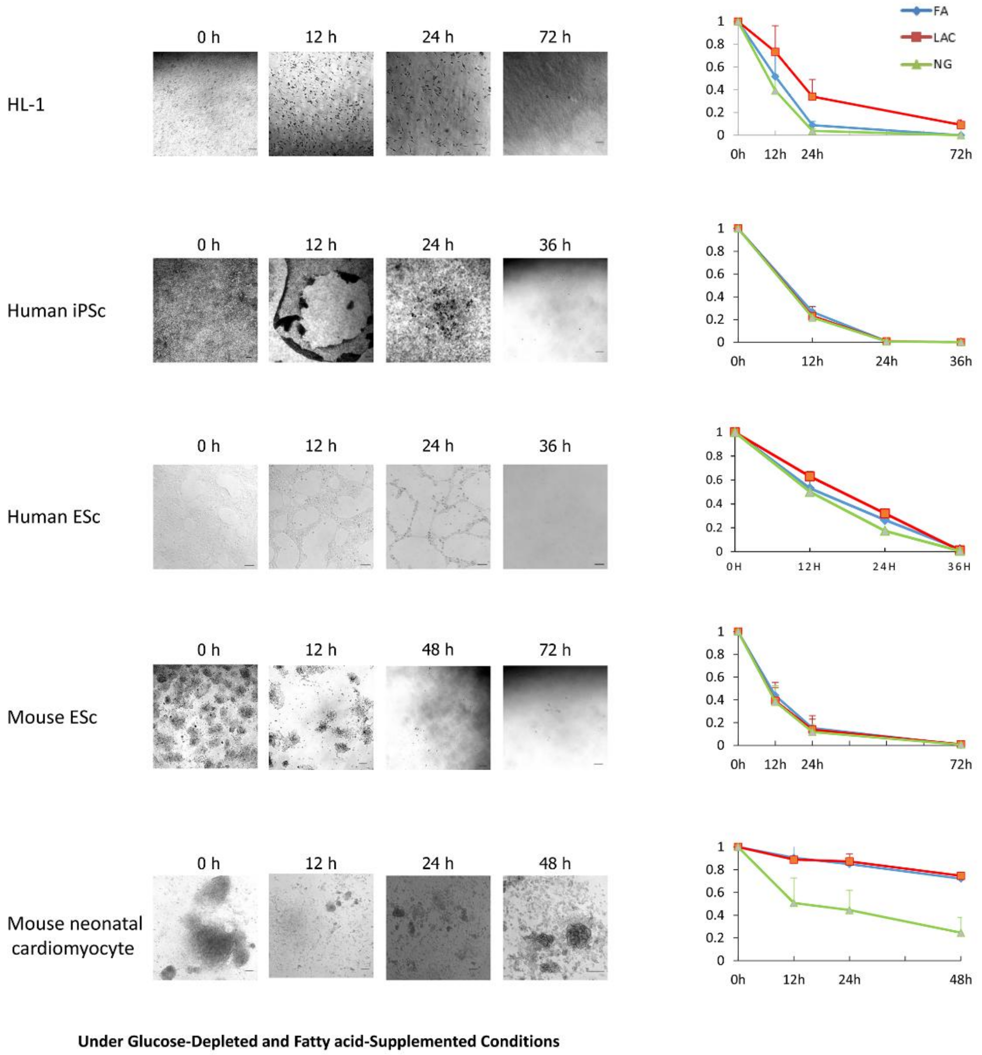

Supplementary Figure 1. Fatty acid-supplemented condition has similar effects as lactate-supplemented condition on cell viabilities of various cell lines. Mouse HL-1 cells, human induced pluripotent stem cells (iPSc), human embryonic stem cells (ESc), mouse embryonic stem cells (ESc) and mouse neonatal cardiomyocytes were cultured with glucose-depleted medium (NG), glucose-depleted medium supplemented with lactate (LAC) and fatty acid (FA). Left panel, representative images of cells under glucose-depleted and fatty acid-supplemented selection. Right panel, time courses of cell viability under glucose-free condition (green lines), glucose-free with lactate-supplemented condition (red lines) and glucose-free with fatty acid-supplemented condition (blue lines) (n=3). The scale bar is 50 μm.

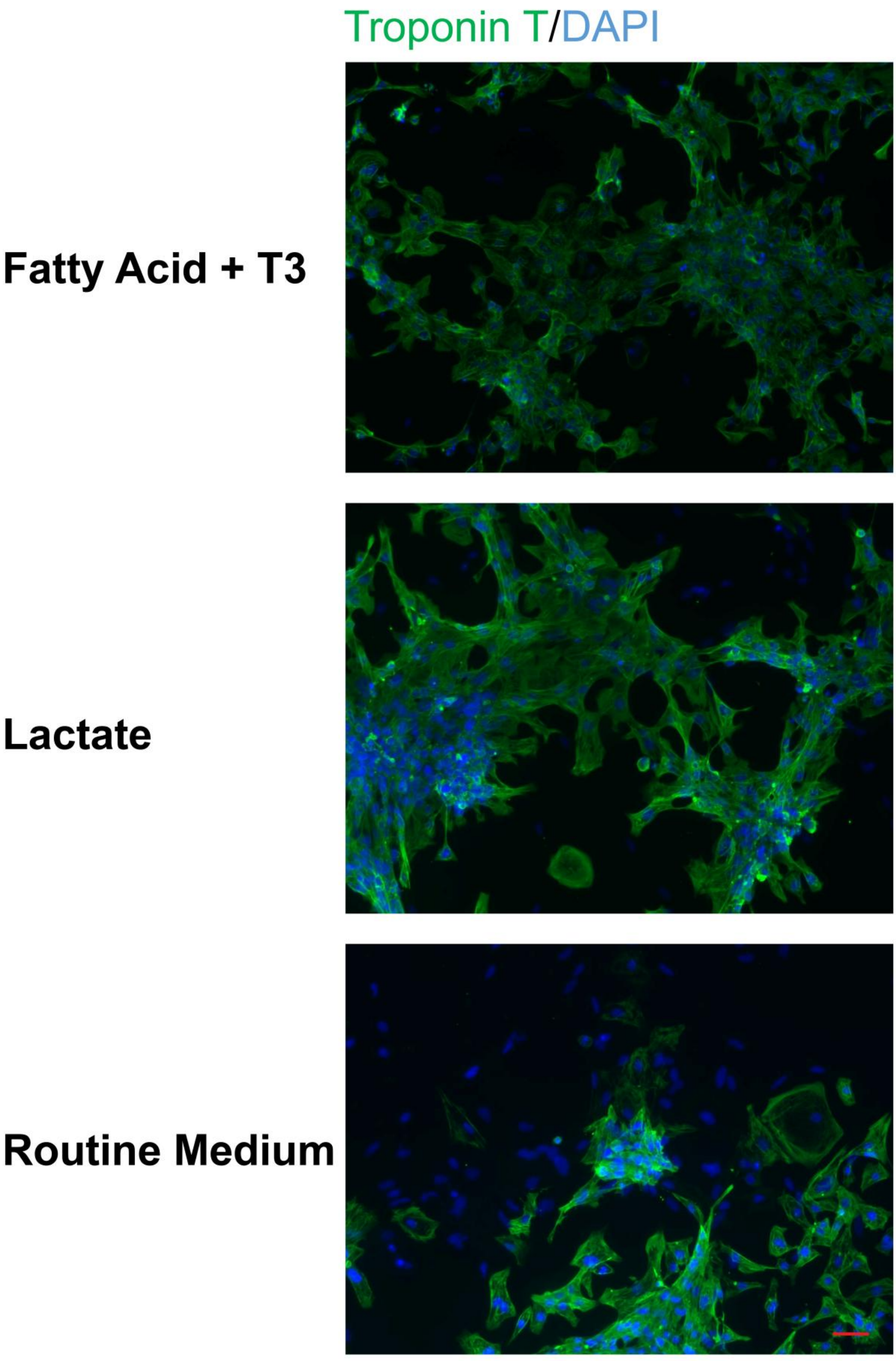

Supplementary Figure 2. Representative photomicrographs showing cardiac Troponin T (green) in hPSC-derived cardiomyocytes cultured in the lactate-supplemented condition, the fatty acid + T_3-supplemented condition, and the

routine medium. Nuclei are stained with DAPI (blue). The red scale bar is 50 μm.

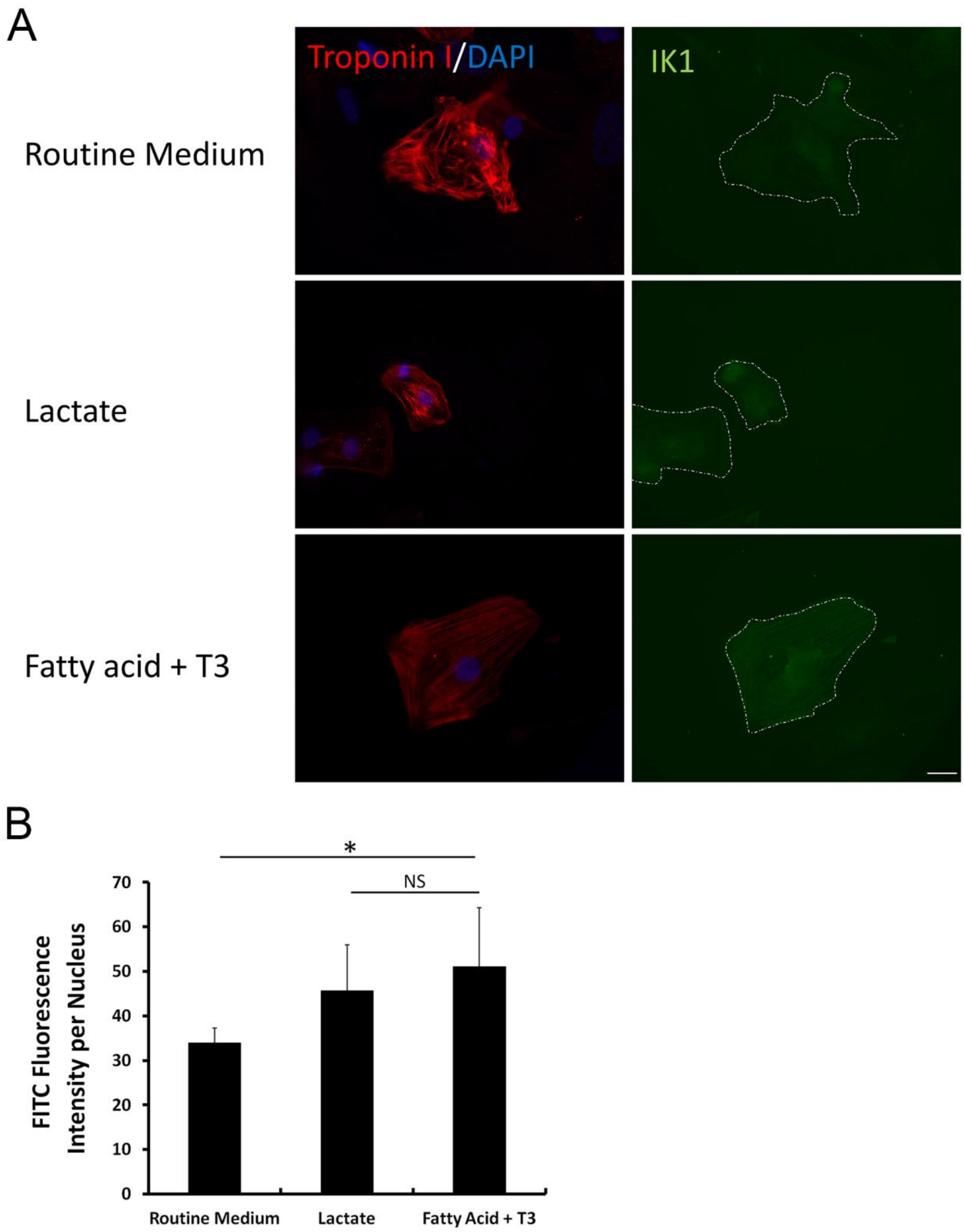

Supplementary Figure 3. The expression of IK1 is up-regulated in fatty acid + T_3-treated cardiomyocytes. (A) Representative images showing IK1 (green) and Troponin I (red) in hPSC-derived cardiomyocytes cultured in the routine medium, lactate-supplemented condition, and fatty acid + T_3-supplemented condition. Nuclei are stained with DAPI (blue). White dash lines outline the cardiomyocytes. The scale bar is 20 μm. (B) Quantification of the FITC fluorescence intensity for each experimental condition indicated in the images. The total intensity was normalized by the numbers of cardiomyocytes (Troponin I-positive cells). n>1000 cells in each

group. NS, no significant difference; *P<0.01.

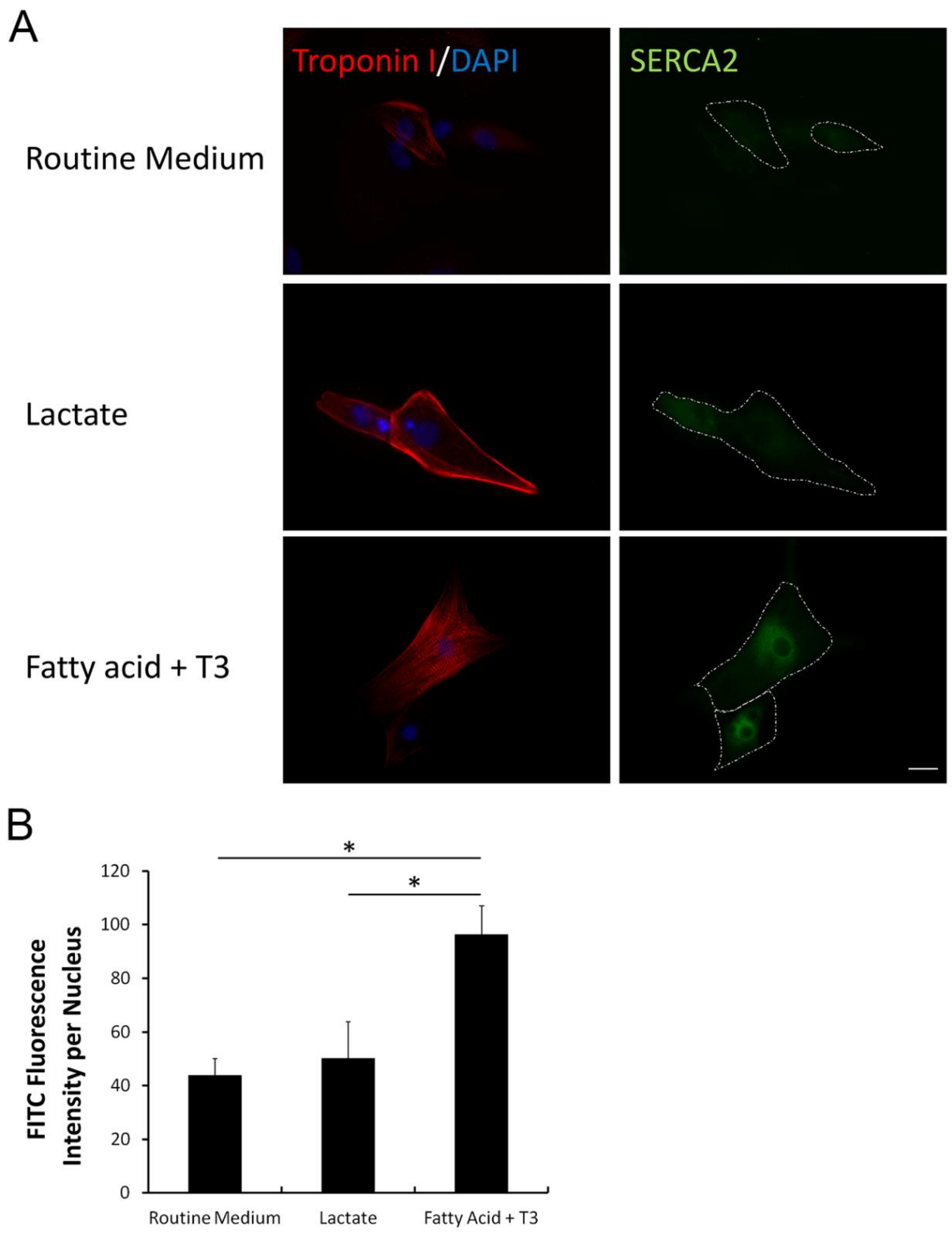

Supplementary Figure 4. The expression of SERCA2 is up-regulated in fatty acid + T_3-treated cardiomyocytes. (A) Representative images showing SERCA2 (green) and Troponin I (red) in hPSC-derived cardiomyocytes cultured in the routine medium, lactate-supplemented condition, and fatty acid + T_3-supplemented condition. Nuclei are stained with DAPI (blue). White dash lines outline the cardiomyocytes. The scale bar is 20 μm. (B) Quantification of the FITC fluorescence intensity for each experimental condition indicated in the images. The total intensity was normalized by the numbers of cardiomyocytes (Troponin I-positive cells). n>1500 cells in each

group. *P<0.01.

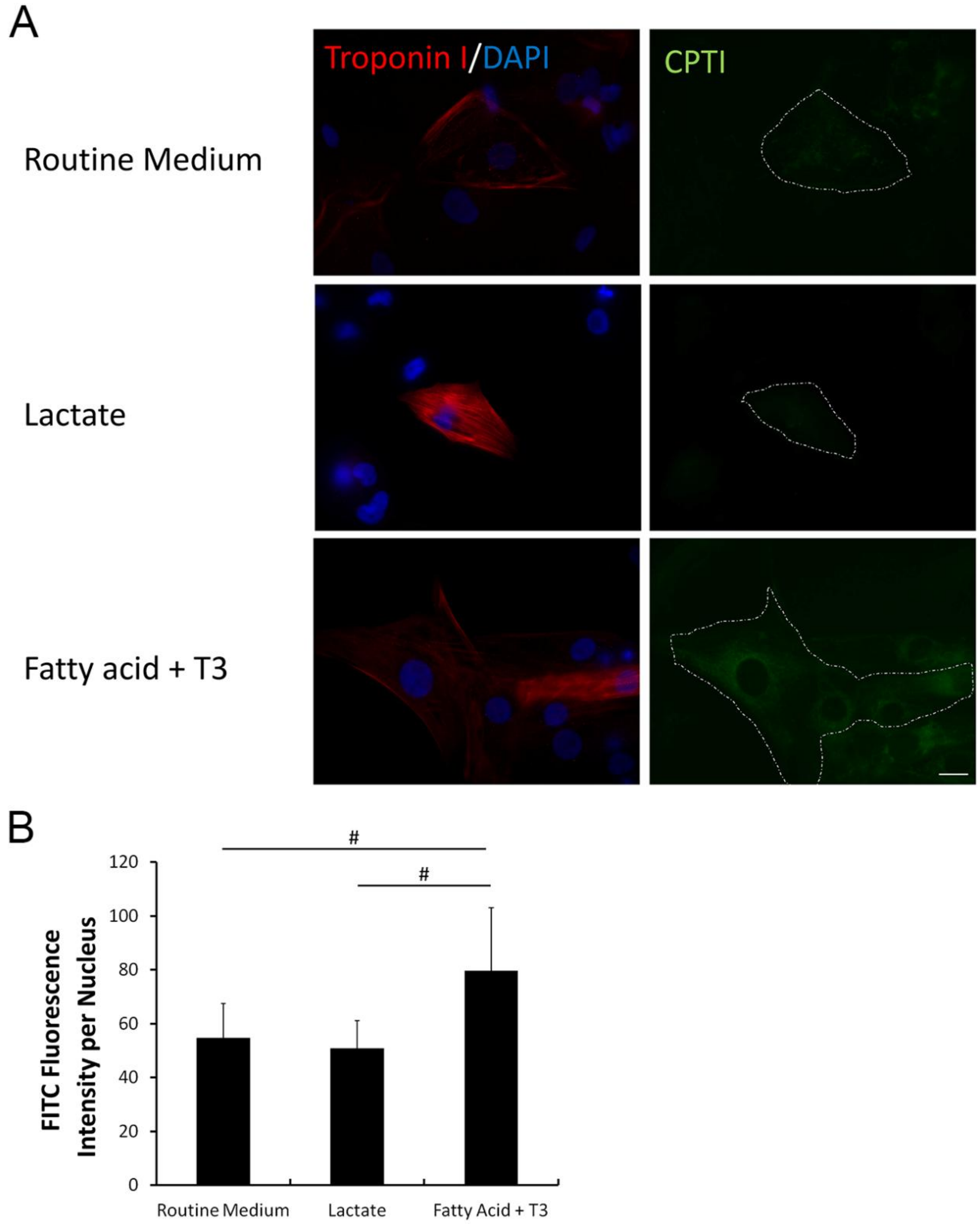

Supplementary Figure 5. The expression of CPTI is up-regulated in fatty acid + T_3-treated cardiomyocytes. (A) Representative images showing CPTI (green) and Troponin I (red) in hPSC-derived cardiomyocytes cultured in the routine medium, lactate-supplemented condition, and fatty acid + T_3-supplemented condition. Nuclei are stained with DAPI (blue). White dash lines outline the cardiomyocytes. The scale bar is 20 μm. (B) Quantification of the FITC fluorescence intensity for each experimental condition indicated in the images. The total intensity was normalized by the numbers of cardiomyocytes (Troponin I-positive cells). n>1900 cells in each

group. #P<0.05.

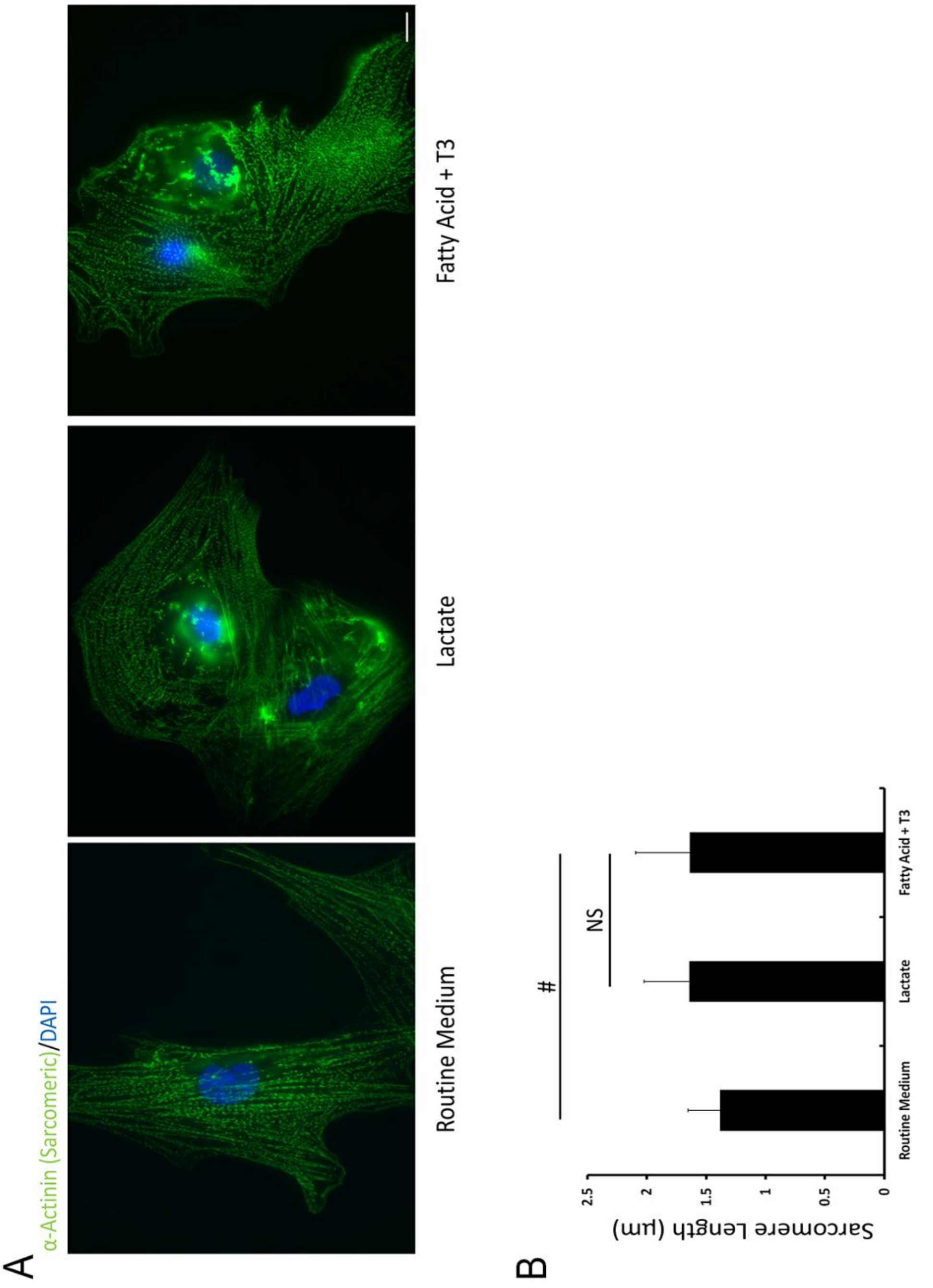

Supplementary Figure 6. The sarcomere length is increased in fatty acid + T_3-treated cardiomyocytes. (A) Representative images showing α-Actinin (green) and nuclei (blue) in hPSC-derived cardiomyocytes cultured in the routine medium, lactate-supplemented condition, and fatty acid + T_3-supplemented condition. The scale bar is 10 μm. (B) Quantification of sarcomere length for each experimental condition. The sarcomere length was measured by the distance between Z-disks. n=30 cells in each group. NS, no significant difference; #P<0.05.

Figure 1C: DAY 3	Routine Medium	Fatty Acid	Lactate	Fatty Acid + T3
Routine Medium	/	*	*	*
Fatty Acid	*	/	#	NS
Lactate	*	#	/	#
Fatty Acid + T3	*	NS	#	/

Figure 1C: DAY 6	Routine Medium	Fatty Acid	Lactate	Fatty Acid + T3
Routine Medium	/	*	*	*
Fatty Acid	*	/	NS	NS
Lactate	*	NS	/	NS
Fatty Acid + T3	*	NS	NS	/

Figure 1C: DAY 8	Routine Medium	Fatty Acid	Lactate	Fatty Acid + T3
Routine Medium	/	*	*	*
Fatty Acid	*	/	NS	NS
Lactate	*	NS	/	NS
Fatty Acid + T3	*	NS	NS	/

Figure 2B	Routine Medium	Fatty Acid	Lactate	Fatty Acid + T3
Routine Medium	/	NS	NS	#
Fatty Acid	NS	/	NS	*
Lactate	NS	NS	/	*
Fatty Acid + T3	#	*	*	/

Figure 2C	Routine Medium	Fatty Acid	Lactate	Fatty Acid + T3
Routine Medium	/	#	#	#
Fatty Acid	#	/	NS	NS
Lactate	#	NS	/	NS
Fatty Acid + T3	#	NS	NS	/

Figure 2D	Routine Medium	Fatty Acid	Lactate	Fatty Acid + T3
Routine Medium	/	NS	NS	#
Fatty Acid	NS	/	NS	NS
Lactate	NS	NS	/	NS
Fatty Acid + T3	#	NS	NS	/

Figure 2E	Routine Medium	Fatty Acid	Lactate	Fatty Acid + T3
Routine Medium	/	*	#	**
Fatty Acid	*	/	**	#
Lactate	#	**	/	**
Fatty Acid + T3	**	#	**	/

Figure 2F	Routine Medium	Fatty Acid	Lactate	Fatty Acid + T3
Routine Medium	/	*	#	**
Fatty Acid	*	/	NS	**
Lactate	#	NS	/	**
Fatty Acid + T3	**	**	**	/

Figure 3B: grey boxes	Routine Medium	Lactate	Fatty Acid + T3	
Routine Medium	/	NS	NS	
Lactate	NS	/	NS	
Fatty Acid + T3	NS	NS	/	

Figure 3B: black boxes	Routine Medium	Lactate	Fatty Acid + T3	
Routine Medium	/	NS	NS	
Lactate	NS	/	NS	
Fatty Acid + T3	NS	NS	/	

Figure 3C: grey boxes	Routine Medium	Lactate	Fatty Acid + T3	
Routine Medium	/	NS	NS	
Lactate	NS	/	NS	
Fatty Acid + T3	NS	NS	/	

Figure 3C: black boxes	Routine Medium	Lactate	Fatty Acid + T3	
Routine Medium	/	NS	**	
Lactate	NS	/	**	
Fatty Acid + T3	**	**	/	

Figure 3E	Routine Medium	Lactate	Fatty Acid + T3	
Routine Medium	/	NS	#	
Lactate	NS	/	#	
Fatty Acid + T3	#	#	/	

Figure 3F	Routine Medium	Lactate	Fatty Acid + T3	
Routine Medium	/	NS	**	
Lactate	NS	/	*	
Fatty Acid + T3	**	*	/	

Figure 4A: *KCNA4*	Routine Medium	Lactate	Fatty Acid + T3	
Routine Medium	/	#	**	
Lactate	#	/	**	
Fatty Acid + T3	**	**	/	

Figure 4A: *KCND3*	Routine Medium	Lactate	Fatty Acid + T3	
Routine Medium	/	NS	**	
Lactate	NS	/	**	
Fatty Acid + T3	**	**	/	

Figure 4A: *KCNQ1*	Routine Medium	Lactate	Fatty Acid + T3	
Routine Medium	/	NS	#	
Lactate	NS	/	#	
Fatty Acid + T3	#	#	/	

Figure 4A: *KCNJ2*	Routine Medium	Lactate	Fatty Acid + T3	
Routine Medium	/	#	*	
Lactate	#	/	*	
Fatty Acid + T3	*	*	/	

Figure 4A: *KCNH2*	Routine Medium	Lactate	Fatty Acid + T3	
Routine Medium	/	#	*	
Lactate	#	/	*	
Fatty Acid + T3	*	*	/	

Figure 4A: *KCNN4*	Routine Medium	Lactate	Fatty Acid + T3	
Routine Medium	/	NS	#	
Lactate	NS	/	NS	
Fatty Acid + T3	#	NS	/	

Figure 4A: *CACNA1C*	Routine Medium	Lactate	Fatty Acid + T3	
Routine Medium	/	*	#	
Lactate	*	/	NS	
Fatty Acid + T3	#	NS	/	
Figure 4A: *SCN5A*	Routine Medium	Lactate	Fatty Acid + T3	
Routine Medium	/	#	#	
Lactate	#	/	#	
Fatty Acid + T3	#	#	/	
Figure 4B: *TNNI3*	Routine Medium	Lactate	Fatty Acid + T3	
Routine Medium	/	*	**	
Lactate	*	/	**	
Fatty Acid + T3	**	**	/	
Figure 4B: *ATP2A2*	Routine Medium	Lactate	Fatty Acid + T3	
Routine Medium	/	*	*	
Lactate	*	/	*	
Fatty Acid + T3	*	*	/	
Figure 4B: *MYH6*	Routine Medium	Lactate	Fatty Acid + T3	
Routine Medium	/	NS	**	
Lactate	NS	/	**	
Fatty Acid + T3	**	**	/	
Figure 4B: *MYH7*	Routine Medium	Lactate	Fatty Acid + T3	
Routine Medium	/	#	**	
Lactate	#	/	**	
Fatty Acid + T3	**	**	/	
Figure 4C: *CPT1A*	Routine Medium	Lactate	Fatty Acid + T3	
Routine Medium	/	#	#	
Lactate	#	/	#	
Fatty Acid + T3	#	#	/	

Figure 4C: *CPT1B*	Routine Medium	Lactate	Fatty Acid + T3	
Routine Medium	/	NS	#	
Lactate	NS	/	#	
Fatty Acid + T3	#	#	/	

Figure 4C: *ACOX1*	Routine Medium	Lactate	Fatty Acid + T3	
Routine Medium	/	#	#	
Lactate	#	/	#	
Fatty Acid + T3	#	#	/	

Figure 4C: *PPARGC1A*	Routine Medium	Lactate	Fatty Acid + T3	
Routine Medium	/	*	*	
Lactate	*	/	#	
Fatty Acid + T3	*	#	/	

Figure 4C: *NRF1*	Routine Medium	Lactate	Fatty Acid + T3	
Routine Medium	/	#	NS	
Lactate	#	/	*	
Fatty Acid + T3	NS	*	/	

Figure 5B	Routine Medium	Lactate	Fatty Acid + T3	
Routine Medium	/	*	*	
Lactate	*	/	NS	
Fatty Acid + T3	*	NS	/	

Figure 5D	Routine Medium	Lactate	Fatty Acid + T3	
Routine Medium	/	**	**	
Lactate	**	/	NS	
Fatty Acid + T3	**	NS	/	

Supplementary Figure 3B	Routine Medium	Lactate	Fatty Acid + T3	
Routine Medium	/	*	*	
Lactate	*	/	NS	
Fatty Acid + T3	*	NS	/	

Supplementary Figure 4B	Routine Medium	Lactate	Fatty Acid + T3	
Routine Medium	/	NS	*	
Lactate	NS	/	*	
Fatty Acid + T3	*	*	/	
Supplementary Figure 5B	Routine Medium	Lactate	Fatty Acid + T3	
Routine Medium	/	NS	#	
Lactate	NS	/	#	
Fatty Acid + T3	#	#	/	
Supplementary Figure 6B	Routine Medium	Lactate	Fatty Acid + T3	
Routine Medium	/	#	#	
Lactate	#	/	NS	
Fatty Acid + T3	#	NS	/	

Supplementary Table 1. The statistical significances between each experimental group in the figures. Statistical significances were evaluated using one-way ANOVA with Bonferroni correction. /, no data; #P<0.05; *P<0.01; **P<0.001; NS, no significant difference.
